# Post-error action control is neurobehaviorally modulated under conditions of constant speeded response

**DOI:** 10.3389/fnhum.2014.01072

**Published:** 2015-01-26

**Authors:** Takahiro Soshi, Kumiko Ando, Takamasa Noda, Kanako Nakazawa, Hideki Tsumura, Takayuki Okada

**Affiliations:** ^1^Department of Forensic Psychiatry, National Institute of Mental Health, National Center of Neurology and PsychiatryKodairo, Japan; ^2^Department of Psychiatry, National Center of Neurology and PsychiatryKodairo, Japan

**Keywords:** error recovery, post-error slowing, cognitive control, impulsivity, Go/No-go paradigm, event-related potentials

## Abstract

Post-error slowing (PES) is an error recovery strategy that contributes to action control, and occurs after errors in order to prevent future behavioral flaws. Error recovery often malfunctions in clinical populations, but the relationship between behavioral traits and recovery from error is unclear in healthy populations. The present study investigated the relationship between impulsivity and error recovery by simulating a speeded response situation using a Go/No-go paradigm that forced the participants to constantly make accelerated responses prior to stimuli disappearance (stimulus duration: 250 ms). Neural correlates of post-error processing were examined using event-related potentials (ERPs). Impulsivity traits were measured with self-report questionnaires (BIS-11, BIS/BAS). Behavioral results demonstrated that the commission error for No-go trials was 15%, but PES did not take place immediately. Delayed PES was negatively correlated with error rates and impulsivity traits, showing that response slowing was associated with reduced error rates and changed with impulsivity. Response-locked error ERPs were clearly observed for the error trials. Contrary to previous studies, error ERPs were not significantly related to PES. Stimulus-locked N2 was negatively correlated with PES and positively correlated with impulsivity traits at the second post-error Go trial: larger N2 activity was associated with greater PES and less impulsivity. In summary, under constant speeded conditions, error monitoring was dissociated from post-error action control, and PES did not occur quickly. Furthermore, PES and its neural correlate (N2) were modulated by impulsivity traits. These findings suggest that there may be clinical and practical efficacy of maintaining cognitive control of actions during error recovery under common daily environments that frequently evoke impulsive behaviors.

## INTRODUCTION

Recovery from error is a self-regulated adjustment in order to prevent future behavioral flaws, which is an adaptive function in normal healthy (NH) individuals. Post-error slowing (PES) is a well-documented action control process immediately after errors (Rabbitt, 1966; Danielmeier and Ullsperger, 2011). PES is a phenomenon where response speed is prolonged after error trials, in order to remedy future errors, and it has been observed in various experimental settings such as those of Flankers (Debener et al., 2005; Cavanagh et al., 2009; Eichele et al., 2010), Simon (King et al., 2010; Danielmeier et al., 2011; Cavanagh et al., 2014), Stop-signal (Li et al., 2008a,b; Lawrence et al., 2009; Chang et al., 2014), and Stroop (Gehring and Fencsik, 2001). PES is associated with several background mechanisms, such as cognitive control, which increases time available to regulate post-error actions (MacDonald et al., 2000; Botvinick et al., 2001; Ridderinkhof et al., 2004), and attentional or affective orienting to rare erroneous events (Hajcak et al., 2003; Notebaert et al., 2009; Wessel et al., 2011).

Neurobehavioral studies show that post-error recovery malfunctions occur in several clinical populations. Liu et al. (2012) reported that patients with obsessive–compulsive disorder (OCD) did not show PES, instead demonstrating shortened post-error response time (RT). Sokhadze et al. (2010) also demonstrated that patients with autism spectrum disorder produced higher commission errors to No-go trials and post-error speeding, rather than demonstrating PES (but for negative evidence, see also Polli et al., 2006; Wild-Wall et al., 2009).

Neuroimaging studies have reported that several cortical areas are associated with PES. The posterior medial prefrontal (pMFC) area, which includes pre-supplementary motor areas (pSMA) and anterior cingulate cortex (ACC), is one of the brain structures most conclusively associated with PES (Garavan et al., 2002; Ridderinkhof et al., 2004; Li et al., 2008a; King et al., 2010; Danielmeier et al., 2011). Danielmeier et al. (2011) observed that the pMFC enhanced hemodynamic responses during error trials, functionally correlated with decreased activity in motor areas (motor inhibition), and was also associated with greater PES. Li et al. (2008b), on the other hand, observed enhanced post-error activation of the ventrolateral prefrontal (PFC) area, which is connected with several areas including the SMA (Ide and Li, 2011).

The pMFC and adjacent medial areas are also likely areas where the event-related cortical potentials (ERPs) associated with PES are generated (Holroyd et al., 1998; Ullsperger and von Cramon, 2001; Garavan et al., 2002; Van Veen and Carter, 2002; Herrmann et al., 2004; Ridderinkhof et al., 2004; Debener et al., 2005; Vocat et al., 2008). Frontocentral-dominant error-related negativity (ERN) or error negativity (Ne) appeared around 100 ms after errors (ERN; Gehring et al., 1993; Ne; Falkenstein et al., 1991). Posterior-dominant error positivity (Pe) follows ERN/Ne (Falkenstein et al., 2000). However, stimulus-locked N200 (N2) responses appear around 200 ms for correct post-error trials (Chang et al., 2014). ERN/Ne, Pe, and N2 are associated with performance monitoring in general (Ridderinkhof et al., 2004; Wessel, 2012), and their larger activities may reflect enhanced monitoring, as demonstrated by their association with greater PES (Gehring et al., 1993; Nieuwenhuis et al., 2001; Hajcak et al., 2003; Debener et al., 2005; Chang et al., 2014).

Unusual ERN/Ne and Pe have been observed in several clinical populations. Hajcak et al. (2008) reported hyperactivation of ERN/Ne for patients with OCD. Additionally, de Bruijn et al. (2006) observed hypoactivation of ERN/Ne in patients with borderline personality disorder (BPD). The patients with BPD also had visually reduced Pe. These findings indicate that hyper and hypo error related neural responses represent abnormal performance monitoring.

It is unclear, on the other hand, whether PES in psychopathology is qualitatively different from PES in NH populations, or whether PES varies with different behavioral traits in NH populations. Impulsivity is one behavioral trait that is likely related to PES. Impulsivity is a multidimensional construct (Patton et al., 1995; Evenden, 1999), defined as rapid, unplanned reactions without regard to negative consequences of behavior (Moeller et al., 2001). Impulsivity is a component of antisocial behaviors such as violence, substance abuse, and suicide (Moeller et al., 2001). Impulsivity is also a symptom of various psychiatric disorders, including antisocial personality disorder (Lijffijt et al., 2012), attention deficit hyperactivity disorder (Winstanley et al., 2006), BPD (Ruchsow et al., 2008), schizophrenia (Hoptman et al., 2014), substance addiction (Li et al., 2006), as well as newly categorized addictive disorders such as internet game addiction (Ding et al., 2014).

Elevated impulsivity may be behaviorally associated with abnormal PES, and also neurally associated with abnormal PFC function, atypical pMFC structures, and deviant error-related ERPs. Chen et al. (2014), for example, examined behavioral inhibition of impulsive violent offenders. In contrast with non-impulsive controls, the impulsive offenders did not show PES. Absence of PES was similarly observed for patients with alcohol dependence and self-reported high impulsivity (Lawrence et al., 2009). Li et al. (2009) also conducted an fMRI experiment concerning response inhibition in impulsive alcohol abuse patients, and observed patients’ decreased activation of the right dorsolateral PFC, which is potentially related to post-error control (Danielmeier et al., 2011). Lee et al. (2013), on the other hand, reported that participants with “ultra high-risk” for psychosis self-reported higher impulsivity than NH controls, and also had reduced grey matter volumes in the rostral ACC, located within the pMFC. Littel et al. (2012) also reported that excessive gamers self-reported higher impulsivity and made more commission errors than NH controls, while simultaneously eliciting reduced ERN/Ne.

Based on previous clinical findings mentioned above, it is hypothesized that impulsivity traits also affect post-error action control under impulsive-like behavioral conditions in healthy populations. Temporal pressure may be one of most suitable conditions to observe varieties of impulsivity traits even in healthy populations (Chen et al., 2008). Speeded response demands may be observed routinely in several situations including video game play, which can be sometimes associated with impulsive traits such as aggression (Hollingdale and Greitemeyer, 2014). Hence, the present study aimed to elucidate how impulsivity traits in healthy people were stimulated, and were associated with post-error monitoring under a speeded response condition.

The present experiment simulated a constant speeded behavioral setting using a Go/No-go task as a popular paradigm investigating response inhibition, and examined ERPs associated with post-error recovery in NH participants. Participants were forced to respond to Go stimuli not only rapidly, but also before stimulus loss occurred (250 ms). Brief stimulus durations were used, similarly to previous studies (Ding et al., 2014), although the present study added the 250 ms time limit task demand on each trial response. These constant speeded conditions may magnify potential individual differences in behavioral inhibition, revealing the relationship between error recovery and impulsivity.

To behaviorally evaluate post-error recovery, we calculated RTs for error No-go trials (ER), pre-error Go trials (PrER), and first and second post-error Go trials (PoER1, PoER2). PES was examined by comparing temporally adjacent post-error trials with the PrER trials (Orr and Hester, 2012). We also calculated RTs for pre-correct Go trials (PrCR), and first and second post-correct Go trials (PoCR1, PoCR2) to examine post-correct slowing (PCS), because PCS and PES may possess different neural and functional foundations (Li et al., 2008b; Ide and Li, 2011; Chang et al., 2014).

To examine neurophysiological correlates of PES, we first examined response-locked ERN/Ne and Pe for the ER trials for error processing. Secondly, we compared stimulus-locked N2 components for the PoER1 and PoER2 trials with the PrER trial in order to examine post-error processing. Finally, N2 components for the PoCR1 and PoCR2 trials were compared with N2 for the PrCR trial to examine post-correct processing. Neurophysiological and behavioral measures were correlated with impulsivity traits which were self-evaluated with the Barrat Impulsiveness Scale (BIS-11; Patton et al., 1995) and the Behavioral Inhibition/Behavioral Activation System scales (BIS/BAS; Carver and White, 1994).

We predicted that the constant speeded response condition would elevate unusual response patterns, delaying PES or yielding post-error speeding even in healthy populations, as observed in OCD (Liu et al., 2012). PES may be more attenuated in peoples with higher impulsivity which more negatively affects post-error control, as suggested by studies of impulsive alcohol abuse patients (Lawrence et al., 2009; Li et al., 2009). Unusual PES may not be associated with error ERPs (in particular, ERN/Ne). Conversely, post-error N2 activities are likely critical for recovery from unusual speeded response patterns (Chang et al., 2014), while also being influenced by impulsivity traits under the present speeded condition.

## MATERIALS AND METHODS

### PARTICIPANTS

Twenty-two NH Japanese participants (14 females, 8 males) were recruited from the community. Their sociodemographic profiles are summarized in **Table 1**. Mean ages of the male and female participants were similar (Mann-Whitney: *U* = 39.0, *p* = 0.267), as were education levels (*U* = 47.5, *p* = 0.570; **Table 1**). The participants’ current psychiatric states were assessed according to SCID-I/NP (Structured Clinical Interview for DSM-IV-TR Axis I Disorders, Non-patient Edition; First et al., 2002) by an experienced psychiatrist or clinical psychologist. Exclusion criteria included current or historical psychiatric illness, brain injury, cognitive impairment, or inability to understand Japanese. Right-handedness was assessed using the Edinburgh handedness inventory (Oldfield, 1971). All participants had normal or corrected to normal vision. Participants provided written informed consent, consistent with the research protocol approved by the Ethical Committee of the National Center of Neurology and Psychiatry (NCNP).

**Table 1:** Demographic and impulsivity characteristics of the participants (*n* = 22).

	Male (*n* = 8)	Female (*n* = 14)	Mann–Whitney

	Mean	SD	Mean	SD	*p*-value
**Demographic**
Age (years)	27	9	32	10	0.267
Education (years)	18	4	16	3	0.570
**BIS-11**
AI	15	3	14	3	0.441
MI	25	5	21	2	0.050
NPI	27	2	25	4	0.145
**BIS/BAS**
BIS	19	5	21	3	0.482
D	12	1	12	2	0.441
RR	17	2	16	2	0.165
FS	12	2	10	2	0.095

*AI, attentional impulsivity; MI, motor impulsivity; NPI, non-planning impulsivity; BIS, behavioral inhibition system; D, drive; RR, reward responsiveness; FS, fun seeking; SD, standard deviation.*

### SPEEDED GO/NO-GO PARADIGM

**FIGURE 1:**
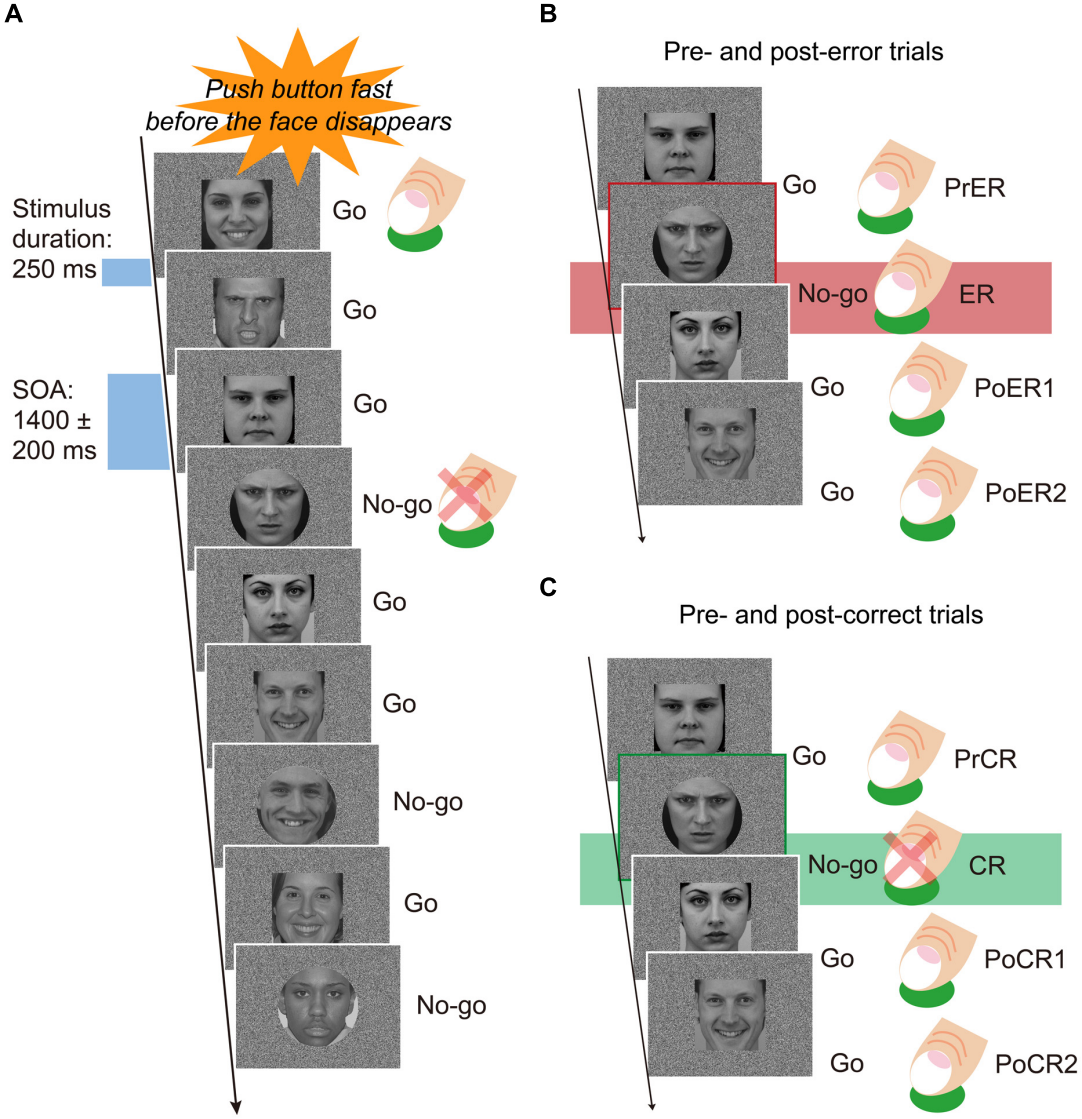
**(A)** Speeded Go/No-go paradigm. Face stimuli were pseudo randomly and successively presented during 250 ms, with a stimulus-onset-asynchrony (SOA) of 1400 ± 200 ms. Participants were forced to press the button as rapidly and correctly as possible before the faces disappeared. One trial block included 120 Go and 60 No-go faces (a total of 180 stimuli). Face stimuli equally included three types of emotional expressions (angry, happy, neutral). In three blocks, rectangle shaped faces were go stimuli, and in another three blocks, circle shaped faces were the Go stimuli. **(B)** A set of trials for post-error analyses. Mean response times (RTs) for pre-error Go trials (PrER), error No-go trials (ER), and first and second post-error Go trials (PoER1, PoER2) were compared to examine post-error processing. Response-locked event-related potentials (ERPs) were calculated for the PrER and ER trials to examine error processing. Stimulus-locked ERPs were derived for the PoER1 and PoER2 trials and compared to the ERP for the PrER trial in order to examine post-error processing. **(C)** A set of trials for post-correct analyses. Mean RTs for pre-correct Go trials (PrCR), first and second post-correct Go trials (PoCR1, PoCR2) were compared for post-correct processing. ERPs were calculated for the PoCR1 and PoCR2 trials, and compared to the ERP for the PrCR trial.

Participants sat on a comfortable chair inside a sound attenuated room, facing a 19-inch display placed 0.9 m in front of their heads. They performed a Go/No-go task under constant speeded response conditions. Participants were instructed to press the Go button as rapidly as possible and also before the response stimuli disappeared (250 ms), using their right index fingers. They were instructed to avoid errors, and the instructions were repeated at least two times before and after practice trials. The stimuli included angry, happy, and neutral faces (**Figure 1A**). Each of the six testing blocks consisted of 180 faces, with equal proportions of the three types of faces (60 angry, 60 happy, 60 neutral), for a total of 180 stimuli. Each block was comprised of 120 Go (about 67%) and 60 No-go (about 33%) faces. Face stimuli were shaped as either rectangles or circles. Go stimuli were equally presented as rectangle or circle shaped faces. Participants were not told that face stimuli included three types of emotional expressions. Face stimuli were presented for 250 ms, and stimulus onset asynchrony was varied at 1400 ± 200 ms. The visual angles of face stimuli were 10.285° vertically and 9.211° horizontally. The order of face stimuli in each block was pseudo randomized so that No-go faces did not appear in succession of two or more. Between four and six Go stimuli always appeared at the beginning of each block. Half of the participants first completed the three blocks with rectangular Go stimuli, and the other half of participants first completed the three blocks with circular Go stimuli. Each block required about 5 min to complete, and the experiment lasted about 35 min, with 30 s rests between testing blocks.

### EXPERIMENTAL STIMULI

Face stimuli were selected from the stimulus sets of the Karolinska Directed Emotional Faces (KDEF: http://www.emotionlab.se/resources/kdef) and the NimStim database (http://www.macbrain.org/resources.htm). Fifteen females and 15 males were selected from each resource, for a total of 60 individuals with each of angry, happy, and neutral facial expressions. A total of 180 face stimuli were converted into gray-scale, and levels of brightness were controlled based on the mean score of 112 (0–255) for the triangle area (40681 pixels) covering eyes, nose, and mouth. Rectangular face stimuli were sized to 330 × 330 pixels, and circular face stimuli were sized to a diameter of 372 pixels, which produced the same size as rectangle shaped face stimuli. Random dot pictures (1158 × 872 pixels) were used as the stimulus background, in order to attenuate rapid visual onset and offset responses and afterimages of the face stimuli. A black fixation cross constantly appeared in the center of the display, except for during the appearance of face stimuli.

### IMPULSIVITY MEASUREMENT

Trait impulsivity was measured with the Japanese version of the self-report questionnaire (Someya et al., 2001), BIS-11 (Patton et al., 1995). BIS-11 contains 30 items clustered into six first order impulsiveness subtraits including attention (No. 5, 9, 11, 20, 28), motor (No. 2–4, 17, 19, 22, 25), self-control (No. 1, 7, 8, 12–14), cognitive complexity (No. 10, 15, 18, 27, 29), perseverance (No. 16, 21, 23, 30), and cognitive instability (No. 6, 24, 26). The BIS-11 also assesses three second order impulsiveness subtraits including attentional (AI: 5, 6, 9, 11, 20, 24, 26, 28; 8–32 scores), motor (MI: 2–4, 16, 17, 19, 21–23, 25, 30; 11–44 scores), and non-planning (NPI: 1, 7, 8, 10, 12–15, 18, 27, 29; 10–40 scores). The BIS-11 is scored using four point Likert scales (4 = very true for me; 3 = somewhat true for me; 2 = somewhat false for me; 1 = very false for me). Subcomponent scores were similar between the male and female participants, with the exception of the MI score (Mann–Whitney: AI, *U* = 44.5, *p* = 0.441; MI: *U* = 27.5, *p* = 0.050; NPI: *U* = 34.0, *p* = 0.145; **Table 1**). Correlation analyses were performed between the second order subtraits (AI, MI, NPI) and the behavioral and neurophysiological measures employed in this study.

Behavioral inhibition characteristics associated with impulsivity (Bari and Robbins, 2013) were also measured by the self-report questionnaire, BIS/BAS scales (Carver and White, 1994). The BIS/BAS is comprised of 20 items, and is answered using four point Likert scales (4 = very true for me; 3 = somewhat true for me; 2 = somewhat false for me; 1 = very false for me). The BIS component (No. 2, 8, 13, 16, 19, 22, 24; 7–28 scores) is associated with avoidance of unpleasant future behavioral consequences. The BAS assesses motivational preference for pleasant behavioral consequences, consisting of drive (D: 3, 9, 12, 21; 4–16 scores), reward responsiveness (RR: 4, 7, 14, 18, 23; 5–20 scores), and fun seeking (FS: 5, 10, 15, 20; 4–16 scores). Our data, obtained using the Japanese version of BIS-11 (Takahashi et al., 2007), resulted in similar scores between male and female participants (BIS: *U* = 45.0, *p* = 0.482; D: *U* = 44.0, *p* = 0.441; RR: *U* = 35.0, *p* = 0.165; FS: *U* = 31.0, *p* = 0.095; **Table 1**). The BIS and three BAS components were used to examine correlations between behavioral traits and neurophysiological responses.

### ELECTROENCEPHALOGRAM RECORDING AND ANALYSES

The electroencephalogram (EEG) epochs (1000 ms before stimulus onset to 1000 ms post-stimulus) for individual trials were recorded from the four midline scalp Ag/AgCl electrodes (frontal: Fz; central: Cz; parietal: Pz; occipital: Oz) with a commercialized EEG system (MEB-2300; NIHON KODEN Corp., Tokyo, Japan). Three electrodes were placed around the eyes for recording horizontal electro-oculogram (HEOG: left-upper minus right-upper) and vertical EOG (VEOG: left-upper minus left-lower). All electrodes were referenced to the linked mastoids. The ground electrode was positioned on participants’ chins. EEGs were recorded at a sampling frequency of 1024 Hz with a band-pass frequency ranging from 0.1 to 100 Hz. The impedance was set below 5000 Ω throughout the experiment.

Stored EEGs were first filtered with a band pass frequency from 0.5 to 40 Hz. VEOG components were reduced from individual epochs by a regression method (Croft and Barry, 2000). Regression coefficients (β) were calculated for EOGs by the regression equation (mEEG = β × VEOG + C; mEEG: measured EEG; C: intercept of the equation). Estimated EEG was calculated by the subtraction equation (estEEG = mEEG – β × VEOG; estEEG: estimated EEG). After EOG reduction, response-locked EEG epochs were obtained for the ER (error No-go) trials and PrER trials from 100 ms before button response (RT) to 350 ms after button response, in order to examine ERN/Ne and Pe. To examine ERPs for post-error processing, stimulus-locked EEG epochs from 100 ms before to 400 ms after the stimulus onset were collected separately for the PrER, PoER1, and PoER2 trials (**Figure 1B**). Concerning ERPs for post-correct processing, epochs with the same duration were collected similarly for the PrCR, PrCR1, and PrCR2 trials (**Figure 1C**). Only complete sets of both pre-/post-error (available epochs: 34 ± 13) and pre-/post-correct (201 ± 20) trials were included in analyses. These analyses did not distinguish between the three emotional conditions, because the mean commission error rate was relatively low (15%). Individual averaged waveforms were calculated after baseline correction (mean potentials during the baseline interval from –100 to 0 ms) and artifact rejection for residual artifacts (peak-to-peak amplitudes of ±75 μV). Mean rejection rates were about 1% for response-locked ERN/Ne and Pe trials, and were about 3% for stimulus-locked ERPs. Grand averaged waveforms were calculated finally.

### STATISTICAL ANALYSES

#### Behavioral performance

We calculated individual commission error rates for No-go trials, and mean RTs for error, pre- and post-error trials (ER, PrER, PoER1, PoER2), and pre- and post-correct trials (PrCR, PoCR1, PoCR2). Omission errors to Go trials were not analyzed because they were absent for almost all participants. RTs faster than 100 ms and slower than 1000 ms were excluded from averaging: a maximum of four RT data points were excluded from each participant, but overall exclusion of data was rare, with zero or one RT data point excluded from almost all participants. Mean RTs were compared using a two-way within-participants ANOVA with factors of No-go trial type (correct, incorrect) and trial order (pre, post1, post2). Pairwise comparisons were conducted among trial orders for each trial type, using Fisher’s Least Significant Difference (LSD) method. Post-error and post-correct RT properties were also represented by the ratios between post- and pre-RTs (post-error: PoER1 or PoER2 : PrER; post-correct: PoCR1 or PoCR2 : PrCR), and were compared to examine their differences in gains of response slowing. Proportional scores greater than 1 represent response slowing and those less than 1 represent response speeding. To examine relationships between behavioral performances and impulsivity traits, Pearson’s correlation coefficients were calculated.

#### Neurophysiological responses

Error-related negativity/Ne and Pe were examined using response-locked waveforms for the ER and PrER trials. ERN/Ne was observed predominantly in frontocentral sites immediately after the button response, and continued for about 160 ms (0–160 ms). Pe appeared immediately after convergence of ERN/Ne (160–350 ms). Two-way within-participants ANOVAs were conducted with response type (correct, error) and electrode (Fz, Cz, Pz, Oz) as factors. When a significant interaction effect appeared, the response type effect was examined for each electrode, using *post hoc* ANOVAs. A Greenhouse-Geisser correction was applied when sphericity was violated. We reported effects related with the response type factor, and described corrected p- and epsilon (ε) values, but unmodified degrees of freedom for easy reference. Finally, we correlated ERP amplitudes (error minus correct) with behavioral performance (error rates and RT ratios) and impulsivity traits, using Pearson’s correlation. ERN/Ne amplitudes in Cz and Pe amplitudes in Pz were used for correlation analyses.

Mean amplitudes for stimulus-locked N2 components for post-error and post-correct processing were compared between the pre-No-go (post-error: PrER; post-correct: PrCR) and post-No-go (post-error: PoER1, PoER2; post-correct: PoCR1, PoCR2) trials. N2 amplitudes were visually specified in comparison with grand average waveforms of error-related trials, because N2 effects were clearly observed during these trials. For the PoER1 trial, the N2 effect was observed predominantly in the frontocentral sites, and was specified as the negative potential deflection between about 120 ms post-stimulus to the end of epoch. For the PoER2 trial, the N2 effect appeared in a similar time window, peaking around 300 ms post-stimulus, predominantly detected by the frontocentral electrodes. Therefore, N2 components for the first and second post-No-go trials were specified equally as the negative deflection during the interval from 120 to 320 ms post-stimulus. Mean N2 amplitudes for the first and second post trials were separately tested for each No-go trial type with two-way within-participants ANOVAs with trial order (pre, post) and electrode (Fz, Cz, Pz, Oz) as factors. We reported effects related with the trial order factor in result sections. We also correlated ERP amplitudes (Fz, Cz) with behavioral performances and impulsivity traits. All of the statistical tests were conducted with SPSS 16.0J (SPSS Inc., Tokyo, Japan).

## RESULTS

### BEHAVIORAL PERFORMANCE

The mean commission error rate for No-go trials was 15.2 ± 6% (mean ± SD), ranging from 4.4 to 30.8% (<10%: 3 persons; 10–20%: 14 persons; >20%: 5 persons). RTs for the PrER, ER, PoER1, and PoER2 trials were 271 ± 28 ms, 266 ± 25 ms, 259 ± 34 ms, and 306 ± 26 ms. RTs for the PrCR, PoCR1, and PoCR2 trials were 281 ± 20 ms, 250 ± 30 ms, and 291 ± 22 ms. RTs were compared using a two-way within-participants ANOVA for No-go trial type and trial order (pre, post1, post2), finding the significant main effect for trial order [*F*_(2,42)_ = 89.131, *p* < 0.0001, ε = 0.792] and the significant interaction [*F*_(2,42)_ = 7.710, *p* = 0.001].

**FIGURE 2:**
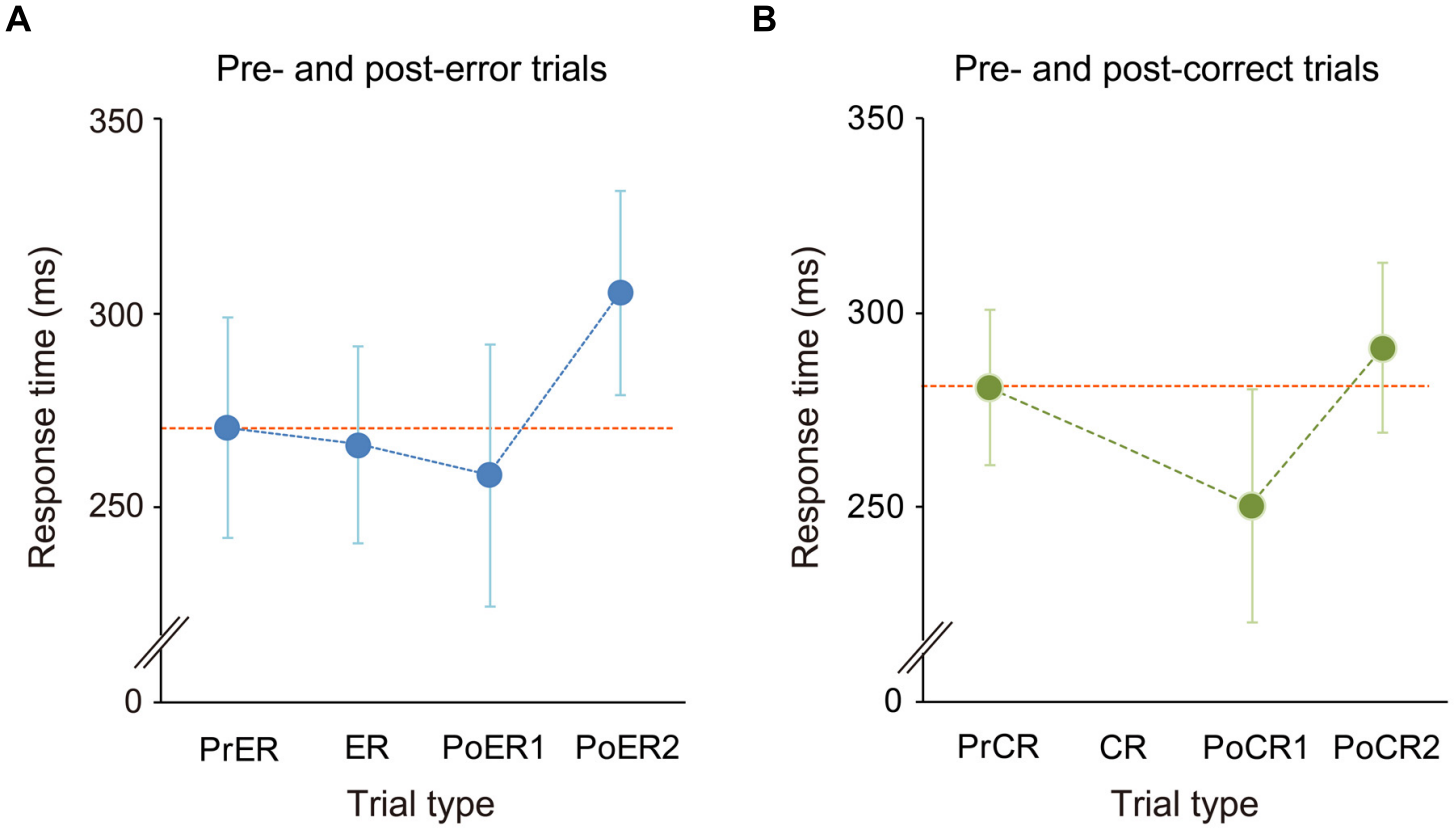
**Response time transitions **(A)** from pre- to post-error trials and **(B)** from pre- to post-correct trials.** Mean RTs for pre-No-go, first and second post trials were compared with the ANOVA with factors of No-go trial type (error, correct) and trial order (pre, post first, post second). Generally, RTs for the first post-No-go trials (PoER1, PoCR1) were faster than those for the pre trials (PrER, PrCR), while RTs for second post trials (PoER2, PoCR2) were slower than those in pre trials. However, response slowing was greater for the PoER2 than the PoCR2. Error bars represent SD of means.

For error-related trials, the RT for the PoER1 trial was shorter than that for the PrER trial, indicating post-error speeding [trial order: *F*_(2,42)_= 42.680, *p* < 0.0001; LSD: PoER1 vs. PrER, *p* = 0.022]. The RT for the PoER2 trial, however, was longer than that for the PrER trial, indicating PES (LSD: PoER2 vs. PrER: *p* < 0.0001; PoER2 vs. PoER1: *p* < 0.0001; **Figure 2A**). For correct-related trials, the RT for the PoCR1 trial was also shorter than that for the PrCR trial, showing post-correct speeding [order: *F*_(2,42)_= 59.951, *p* < 0.0001; LSD: PoER1 vs. PrER, *p* < 0.0001]. In contrast, the PoCR2 needed longer RT than the PrCR, showing PCS (LSD: PoER2 vs. PrER: *p* < 0.0001; PoER2 vs. PoER1: *p* < 0.0001; **Figure 2B**). Therefore, irrespective of response accuracy, response speeding occurred for Go trials immediately after No-go trials, and post slowing occurred late after speeding.

Follow-up analyses for comparisons between error- and correct-related trials demonstrated that the PrER trial was responded more rapidly than the PrCR trial (LSD: *p* = 0.006), and the PoER2 trial needed longer RT than the PoCR2 trial (*p* < 0.0001), which suggests that response slowing was larger for post-error than post-correct trials. This was also supported by direct comparison of RT ratios (Post2 : Pre): the ratio of the PoER2 trial (1.15 ± 0.1) was significantly higher than that of the PoCR2 trial [1.04 ± 0.02; *t*_(1,21)_ = 5.763, *p* < 0.0001].

**FIGURE 3:**
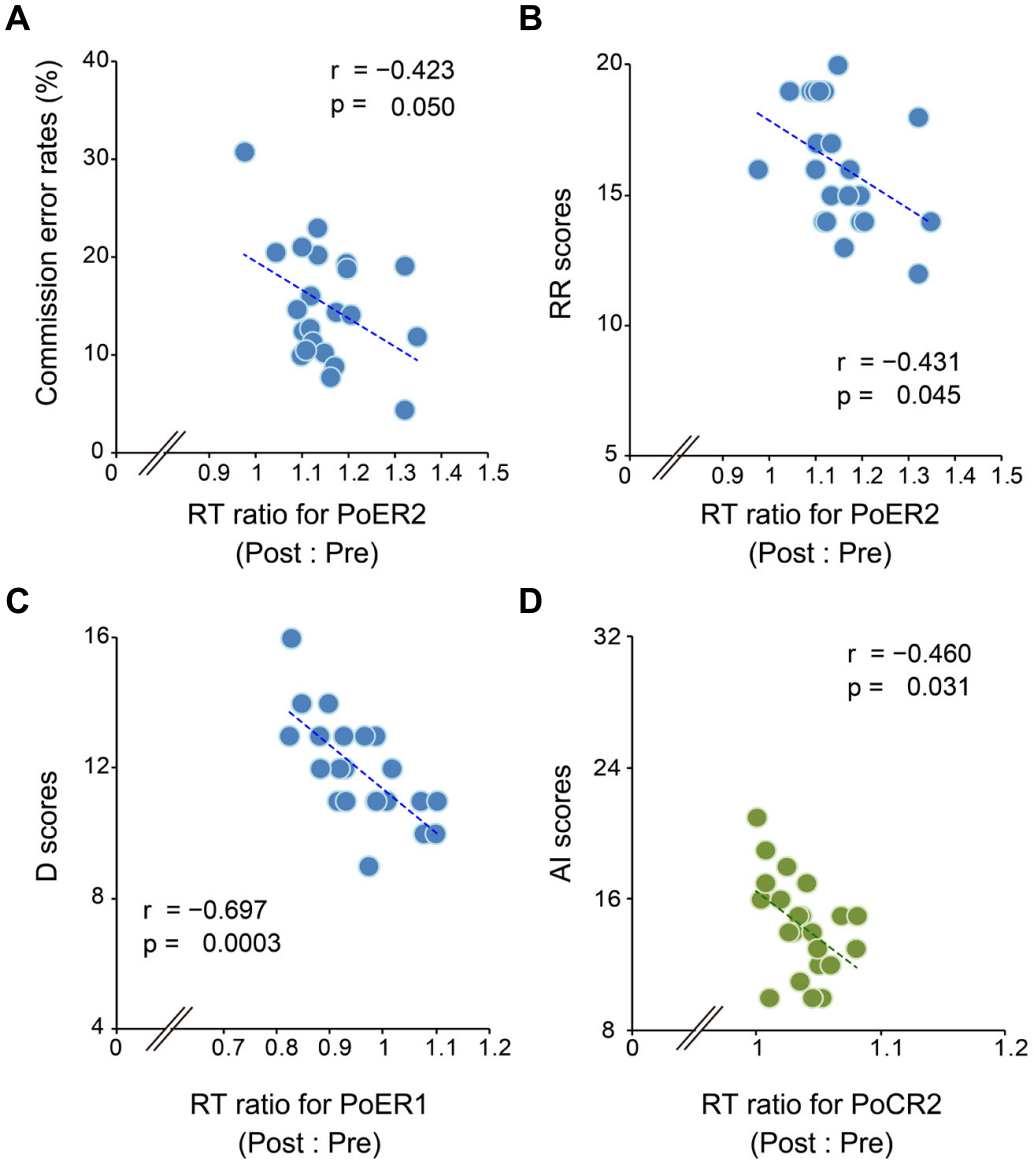
**Correlations between response slowing properties and other behavioral measures.** Response slowing is represented by the ratio between RTs for post- and pre-No-go trials (post : pre). Scores greater than 1 represent response slowing. **(A)** Almost all RT ratios for the PoER2 trial are greater than 1 (post-error slowing, PES), and negatively correlated with commission error rates. **(B)** RT ratios for the PoER2 trial are negatively correlated with the reward responsiveness (RR). **(C)** Almost all RT ratios for the PoER1 trial are less than 1 (post-error speeding), and are negatively correlated with the drive (D) trait. **(D)** Almost all RT ratios for the PoCR1 trial are greater than 1 (post-correct slowing, PCS), and are negatively correlated with attentional impulsivity (AI).

Correlational analyses between error rates and post-error RT ratios revealed that greater PES at the PoER2 trial was associated with smaller error rates (*r* = –0.423, *p* = 0.050; **Figure 3A**), indicating that PES functions in a compensatory way to reduce errors. The RT ratio for the PoER2 trial was also negatively correlated with RR (*r* = –0.431, *p* = 0.045; **Table 2**), indicating that higher RR traits were associated with attenuated PES (**Figure 3B**). The RT ratio for the PoER1 trial was negatively correlated with the D trait (*r* = –0.697, *p* = 0.0003; **Table 2**), showing that higher drive traits were associated with greater post-error speeding (**Figure 3C**).

**Table 2:** Correlation coefficients between behavioral performances and impulsivity traits (*n* = 22).

	Error rate	RT ratio for PoER1	RT ratio for PoER2	RT ratio for PoCR1	RT ratio for PoCR2
**BIS-11**
AI	0.163	0.065	-0.032	-0.314	-0.460*
MI	0.209	-0.270	-0.272	-0.265	-0.085
NPI	0.049	-0.068	0.037	-0.203	-0.316
**BIS/BAS**
BIS	0.073	-0.035	-0.083	-0.099	-0.114
D	0.238	-0.697***	-0.411	-0.022	0.224
RR	0.176	-0.368	-0.431*	-0.102	-0.097
FS	0.032	-0.179	-0.354	-0.163	0.127

RT, response time; PoER1, first post-error Go trials; PoER2, second post-error Go trials; PoCR1, first post-correct Go trials; PoCR2, second post-correct Go trials; AI, attentional impulsivity; MI, motor impulsivity; NPI, non-planning impulsivity; D, drive; RR, reward responsiveness; FS, fun seeking. *p < 0.05; ***p < 0.001. Scores represent Pearson’s correlation coefficients.

On the other hand, correlations between error rates and post-correct RT ratios did not reach significance (PoCR1: *r* = –0.010, *p* = 0.964; PoCR2: *r* = –0.062, *p* = 0.784). The RT ratio for the PoCR2 trial was negatively correlated with AI (*r* = –0.460, *p* = 0.031; **Table 2**), demonstrating that higher AI traits were associated with smaller PCS (**Figure 3D**).

### NEUROPHYSIOLOGICAL RESPONSES

#### Response-locked ERPs for error No-go trials

**FIGURE 4:**
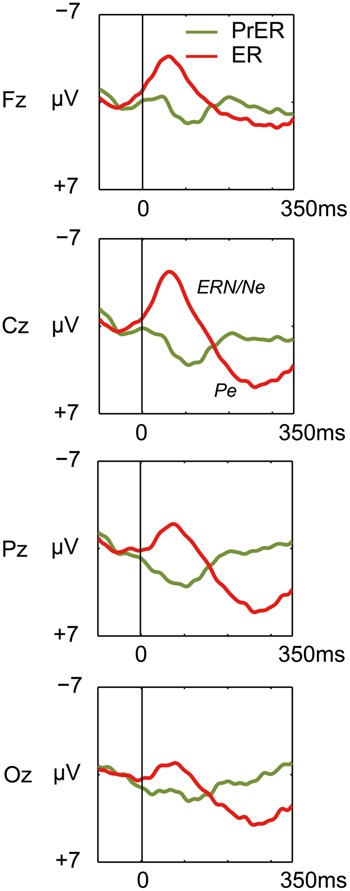
**Comparison of response-locked ERP waveforms for pre-error correct Go trial (PrER: green) and ER (red) at four midline electrodes (Fz, Cz, Pz, Oz).** Negative voltage (μV) is plotted upwardly. The baseline for ERP comparison is the interval from 100 ms pre-stimuli to stimulus onset. Frontocentral error negativity (ERN/Ne) and posterior-dominant error positivity (Pe) are clearly observed.

Error-related negativity/Ne and Pe for the ER trials were tested with two-way ANOVAs with response type (correct, incorrect) and electrode (Fz, Cz, Pz, Oz) as factors. The ANOVA for ERN/Ne showed a significant main effect for response type [*F*_(1,21)_= 103.098, *p* < 0.0001] and a response type × electrode interaction [*F*_(3,63)_ = 22.731, *p* < 0.0001, ε = 0.556]. ERN/Ne was significantly observed for all electrodes [*F_s_*_(1,21)_> 24.691, *p_s_* < 0.001; **Figure 4**]. The ANOVA for Pe showed a significant main effect for response type [*F*_(1,21)_= 29.287, *p* < 0.0001] and a response type × electrode interaction [*F*_(3,63)_ = 12.856, *p* < 0.0001, ε = 0.703]. *Post hoc* ANOVAs showed that Pe was significantly different in all comparisons [*F_s_*_(1,21)_> 5.20, *p_s_* < 0.033; **Figure 4**].

Error-related negativity/Ne (Cz) and Pe (Pz) were not correlated with the post-error and post-correct RT ratios (**Table 3**). Neither type of ERP was correlated with impulsivity scores, but ERN/Ne showed a trend toward being significantly correlated with NPI (*r* = –0.413, *p* = 0.056; **Table 4**).

**Table 3:** Correlation coefficients between ERP amplitudes and behavioral performances (*n* = 22).

	ER		PoER1		PoER2		PoCR1		PoCR2	
	ERN/Ne	Pe	Fz	Cz	Fz	Cz	Fz	Cz	Fz	Cz
Error rate	–0.063	–0.261	0.172	0.232	0.259	0.079	–0.105	0.044	0.203	0.197
RT ratio for PoER1	0.076	0.413	–0.156	–0.260	–	–	–	–	–	–
RT ratio for PoER2	0.155	0.190	–	–	–0.623**	–0.568**	–	–	–	–
RT ratio for PoCR1	–0.236	0.047	–	–	–	–	–0.040	–0.245	–	–
RT ratio for PoCR2	0.019	0.305	–	–	–	–	–	–	–0.019	–0.085

ER, error trials; ERN/Ne, error-related negativity/error negativity; Pe, error positivity; PoER1, first post-error Go trials; PoER2, second post-error Go trials; PoCR1, first post-correct Go trials; PoCR2, second post-correct Go trials; RT, response time; **p < 0.01. Scores indicate Pearson’s correlation coefficients.

**Table 4:** Correlation coefficients between ERP amplitudes and impulsivity traits (*n* = 22).

	ER		PoER1		PoER2		PoCR1		PoCR2	
	ERN/Ne	Pe	Fz	Cz	Fz	Cz	Fz	Cz	Fz	Cz
**BIS-11**
AI	0.229	–0.235	0.032	0.109	0.171	0.058	0.279	0.317	–0.224	–0.156
MI	–0.207	–0.368	–0.559**	–0.445*	0.344	0.309	0.322	0.297	0.081	0.027
NPI	–0.413	–0.221	–0.053	–0.026	0.246	0.184	0.389	0.485*	–0.063	0.041
**BIS/BAS**
BIS	0.021	–0.128	–0.123	–0.066	0.339	0.453	0.312	0.380	–0.408	–0.337
D	–0.122	–0.373	0.228	0.327	0.412	0.319	0.051	0.070	–0.105	–0.072
RR	–0.007	–0.369	–0.188	–0.082	0.706**	0.569**	0.371	0.383	–0.304	–0.198
FS	0.235	–0.206	–0.004	0.088	0.453*	0.271	0.225	0.246	–0.161	–0.108

ER, error trials; ERN/Ne, error-related negativity/error negativity; Pe, error positivity; PoER1, first post-error Go trials; PoER2, second post-error Go trials; PoCR1, first post-correct Go trials; PoCR2, second post-correct Go trials; AI, attentional impulsivity; MI, motor impulsivity; NPI, non-planning impulsivity; D, drive; RR, reward responsiveness; FS, fun seeking; *p < 0.05; **p < 0.01. Scores represent Pearson’s correlation coefficients.

#### Stimulus-locked ERPs for post-error Go trials

**FIGURE 5:**
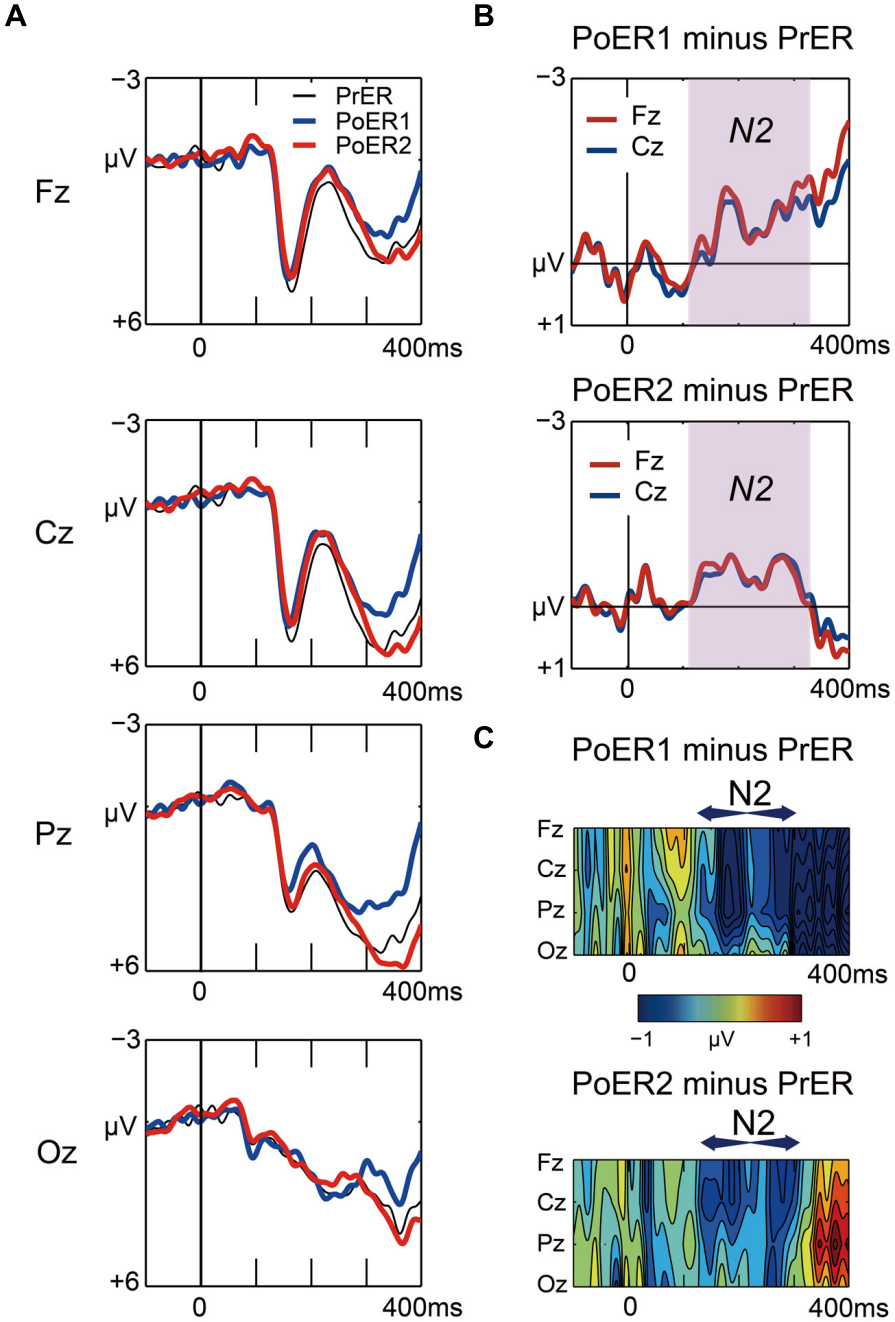
**(A)** Comparisons of stimulus-locked ERP waveforms for pre-error (black line for PrER) and first and second post-error (blue line for PoER1, red line for PoER2) Go trials at four midline scalp electrodes (Fz, Cz, Pz, Oz). Negative voltage (μV) is plotted upwardly. The baseline for ERP comparison is the interval from 100 ms pre-stimuli to stimulus onset. **(B)** Difference waveforms (post minus pre) at frontocentral electrodes are plotted for the PoER1 trial in the top graph, and for the PoER2 trial in the bottom graph. For the PoER1 trial, the N2 effect appears from around 120 ms (shades of pink). For the PoER2 trial, N2 is also observed in the pink shaded time interval. **(C)** Spatiotemporal mapping of difference potentials (post minus pre) for the PoER1 trial in the top graph and for the PoER2 trial in the bottom graph. Blue areas represent negative potential enhancement and red areas represent positive potential enhancement. The horizontal axis indicates the temporal range from 100 ms pre-stimulus to 400 ms post-stimulus, and the vertical axis represents the spatial expansion from frontal to occipital scalp areas.

Grand average waveforms for the PrER (black), PoER1 (blue), and PoER2 (red) trials are plotted in **Figure 5A**. For ease of visual inspection of ERP effects, difference waveforms (post-error minus pre-error) and spatiotemporal maps of subtraction potentials are represented in **Figures 5B,C**, respectively. Negative voltages for the PoER1 trial and for the PoER2 trial were enhanced compared to the PrER trial during the N2 time window (120–320 ms). Accordingly, mean amplitudes during this interval were compared between post-error trials and the PrER trial.

**FIGURE 6:**
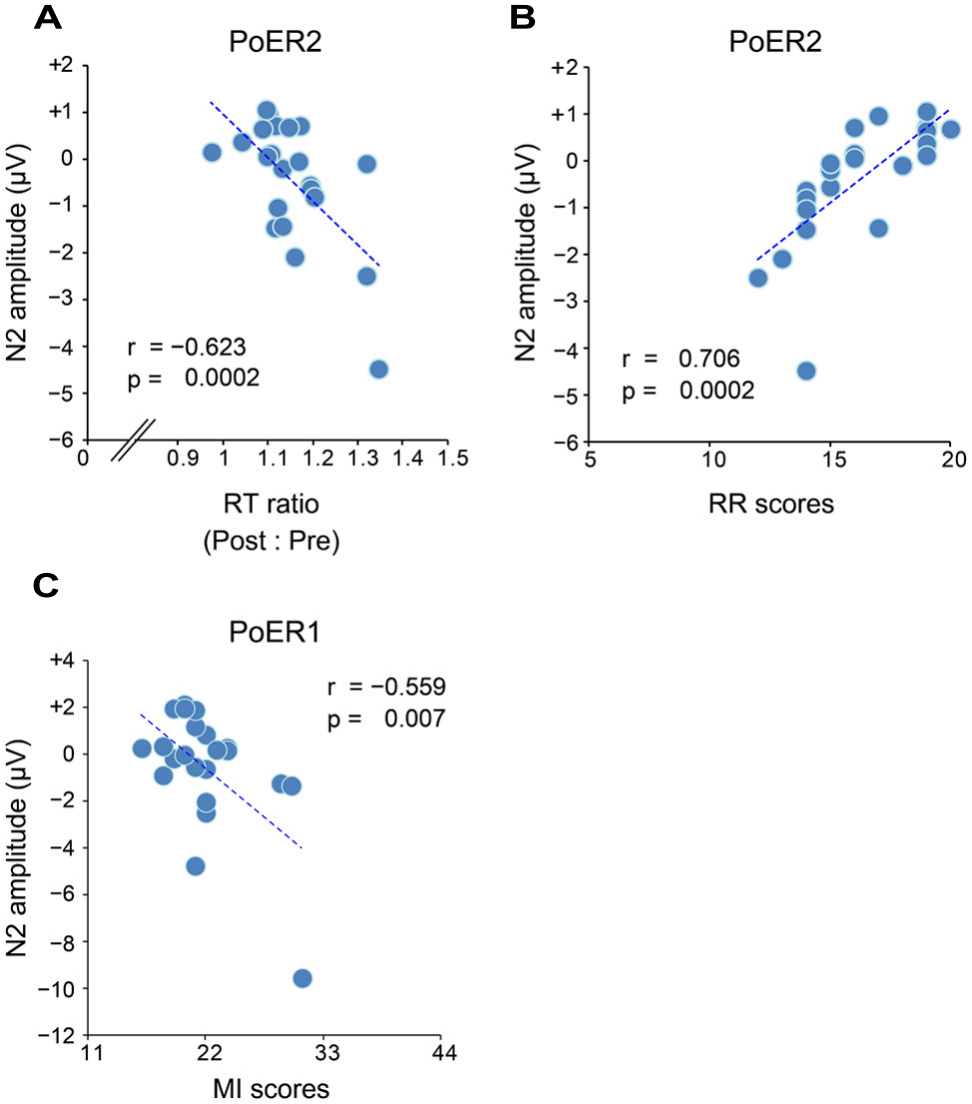
**(A)** Negative correlation between PES and N2 (Fz) for the second post-error Go trial (PoER2). PES is represented by the ratio between post- and pre-error RTs. Scores greater indicate larger PES. **(B)** Positive correlation between N2 (Fz) for the PoER2 trial and reward responsiveness (RR). **(C)** Negative correlation between N2 (Fz) for the first post-error Go trial (PoER1) and motor impulsiveness (MI).

For the PoER1 trial, a two-way ANOVA did not show significant effects [trial order: *F*_(1,21)_ = 1.752, *p* = 0.2; trial order × electrode:* F*_(3,63)_ = 1.691, *p* = 0.199, ε = 0.614]. The N2 effect also did not reach significance for the PoER2 trial [trial order: *F*_(1,21)_ = 1.210, *p* = 0.284, trial order × electrode: *F*_(3,63)_ = 1.456, *p* = 0.246, ε = 0.557]. Approximately 50% of the participants (*n* = 10) yielded positive effects at the PoER2 trial (e.g., amplitudes at Fz: 0.56 ± 0.35 μV), in contrast with other participants (*n* = 12) with negative effects (–1.27 ± 1.27 μV), indicating that N2 activities may vary with individual differences in behavioral performances and/or traits.

Correlational relationships were first examined between N2 amplitudes and behavioral performances. The output coefficients are summarized in **Table 3**. N2 for the PoER2 trial was negatively correlated with the RT ratio for the PoER2 trial (Fz: *r* = –0.623, *p* = 0.0002; Cz: *r* = –0.568, *p* = 0.006), showing that greater (more negative) N2 activity was associated with greater PES (**Figure 6A** for Fz). This correlation was also significant when age and sex were controlled [Fz: *r_xy⋅z_* (partial correlation coefficient) = –0.588, *p* = 0.006; Cz: *r_xy⋅z_* = –0.50, *p* = 0.025]. N2 for the PoER1 trial was not significantly correlated with the RT ratio for the PoER1 trial. N2 components for both post-error trials were not significantly correlated with commission error rates.

Correlations between N2 amplitudes and impulsivity traits were examined similarly to the analyses described above. The results are summarized in **Table 4**. N2 for the PoER2 trial was positively correlated with the BIS and RR scores (BIS: Cz, *r* = 0.453, *p* = 0.034; RR: Fz, *r* = 0.706, *p* = 0.0002; Cz, *r* = 0.569, *p* = 0.006). The positive correlation between N2 and RR remained significant when age and sex were controlled (Fz: *r_xy⋅z_* = 0.535, *p* = 0.015), showing that greater N2 activity in the PoER2 trial was associated with less reward impulsivity (**Figure 6B** for Fz). However, N2 for the PoER1 trial was negatively correlated with MI (Fz: *r* = –0.559, *p* = 0.007; Cz: *r* = –0.445, *p* = 0.038; **Figure 6C** for Fz). This correlation remained significant after statistical removal of age and sex effects (Fz: *r_xy⋅z_* = –0.532, *p* = 0.016), reconfirming that elevated motor impulsivity is conversely associated with greater N2 activities immediately after errors.

#### Stimulus-locked ERPs for post-correct Go trials

**FIGURE 7:**
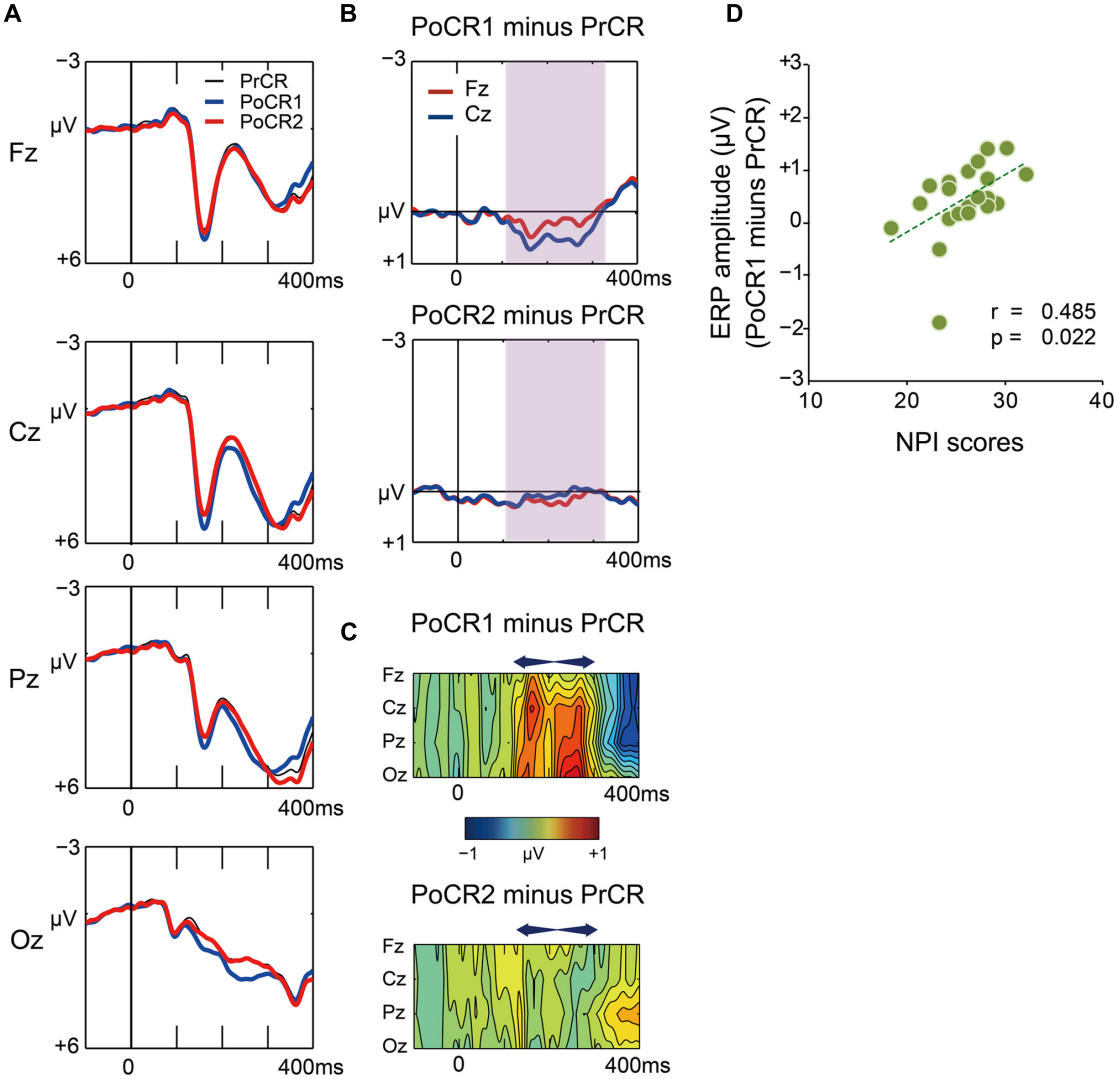
**(A)** Comparisons of ERP waveforms for pre-correct (black line for PrCR) and first and second post-correct (blue line for PoCR1; red line for PoCR2) Go trials at midline scalp electrodes. Negative voltage (μV) is plotted upwardly. The baseline for comparison is the interval from 100 ms pre-stimuli to stimulus onset. **(B)** Frontocentral difference waveforms (post minus pre) are plotted for the PoCR1 trial in the top graph, and for the PoCR2 trial in the bottom graph. For the PoCR1 trial, the reduced N2 (positive) effect appears from around 120 ms (shades of pink). For the PoCR2 trial, ERP effect is not clearly observed. **(C)** Spatiotemporal mapping of difference potentials (post minus pre) for the PoER1 trial in the top graph and for the PoER2 trial in the bottom graph. Blue areas represent negative potential effects and red areas represent positive effects. The horizontal axis indicates the temporal range from 100 ms pre-stimulus to 400 ms post-stimulus, and the vertical axis represents the electrode locations. **(D)** Positive correlations between ERP amplitudes (post minus pre) for the PoCR1 trial and non-planning impulsivity (NPI).

Grand average waveforms for the PrCR (black), PoCR1 (blue), and PoCR2 (red) trials are plotted in **Figure 7A**. Difference waveforms (post-correct minus pre-correct) and spatiotemporal maps of subtraction potentials are represented in **Figures 7B,C**, respectively. Contrary to post-error trials, the PoCR1 and PoCR2 trials did not enhance negative voltages during the N2 time window (120–320 ms). Conversely, the PoCR1 yielded positive effects.

For the PoCR1 trial, a two-way ANOVA showed a significant main effect for trial order [trial order: *F*_(1,21)_ = 15.917, *p* = 0.001; trial order × electrode:* F*_(3,63)_ = 2.271, *p* = 0.089], supporting the visual inspection of the positive effect for the PoCR1 trial. The ANOVA for the PoCR2 trial did not observe significance effects related with trial order [trial order: *F*_(1,21)_ = 0.818, *p* = 0.376; trial order × electrode: *F*_(3,63)_ = 0.527, *p* = 0.563, ε = 0.556].

Correlations were similarly examined between ERPs and behavioral performances. The results are summarized in **Table 3**. Frontocentoral amplitudes for the PoCR1 and PoCR2 trials were not significantly correlated with the error rates and RT ratios.

Correlations between ERP amplitudes and impulsivity traits were examined finally. The results are summarized in **Table 4**. ERP amplitudes for the PoCR1 trial were positively correlated with the NPI scores (Cz: *r* = 0.485, *p* = 0.022; *r_xy⋅z_* = 0.450, *p* = 0.047; **Figure 7D**). Although significant correlations were not obtained for the MI scores, the positive coefficient between ERPs and the MI was observed descriptively for the PoCR1 trial (Fz: *r* = 0.320). These results suggest that cortical activities for the PoCR1 trial may be different from those for the PoER1 trial, which were negatively correlated with the MI in a reverse manner (Fz: *r* = –0.559).

## DISCUSSION

Humans develop a high-loaded performance monitoring system, mainly located in the pMFC, in order to prevent future behavioral flaws. While such an adaptive monitoring system sometimes malfunctions in people with pathological impulsivity, it is still unclear how error recovery is affected by impulsivity traits in NH people. The present study simulated a constant speeded response condition, and examined post-error action control in NH participants, using ERPs. We predicted that the temporal pressure would promote individual differences in impulsivity, and post-error recovery and related neural activities would change with impulsivity scores. Firstly, we found that PES did not take place immediately after errors, but rather, we observed post-error speeding and later PES. The greater PES was associated with smaller commission errors and less impulsivity. However, the significant correlation between error rates and PCS was not observed. Secondly, greater PES was associated with enhanced N2 activity at the second post-error trials, which in turn, was associated with less impulsivity. Such neurobehavioral correlation did not appear at the second post-correct trial which showed PCS. Contrary to previous studies, error-related ERPs were not significantly correlated with PES.

### DELAYED POST-ERROR SLOWING

Participants did not immediately recover from rash response patterns in the PoER1 trial, as demonstrated by their faster responses compared with those in the PrER trial. PES occurred late during the PoER2 trial. Such delayed post-error recovery has not been observed in previous studies examining RT changes during multiple PoER trials (Eichele et al., 2010). While Notebaert et al. (2009) reported that frequent (75%) errors tended to yield post-error speeding in contrast to PES after infrequent correct responses, the error events in the present study occupied at most 15% of the total. Therefore, frequency may not be irrelevant to post-error speeding during the PoER1 trial.

One possible interpretation of the present post-error recovery pattern is that the speeded response condition promoted affective excitement (Horn et al., 2003), which resulted in participants continuing to involuntarily make risky, speeded responses, potentially causing subsequent errors. The post-error RT property at the PoER1 trial was negatively correlated with the drive trait associated with excitement: larger post-error speeding was associated with higher drive traits. This suggests that reversing of induced excitement did not occur quickly during the PoER1 trial because of this enhanced drive behavioral property, and therefore, PES did not appear at the PoER1 trial.

Another possible interpretation is that the present task setting enhanced affective arousal, negatively influencing post-error recovery. Experimental observations showed that many participants made an exclamation of surprise in response to their own errors during the practice trials, despite being strictly instructed not to do so. Such affective arousal may evoke absentmindedness (that is, an out of control state) correlated with impulsivity and ACC activity (Garavan et al., 2002). Accordingly, recovery from an accelerated response pattern may happen late in the PoER2 trial, while the extent of PES was still influenced by the affective trait of RR.

This interpretation seems to be consistent with PCS observed in the second post-correct trial. Although response slowing was also observed in the PoCR2 trial, PCS was significantly correlated with attentional impulsivity (AI), rather than affective impulsivity. This contrast between PES and PCS may come from their differences in affective arousal. Affective arousal may appear after inhibition failure more saliently than successful inhibition, and hence, PES might be significantly associated with affective impulsivity under the present speeded condition.

Although PES was delayed in the present study, a significant correlation was observed between PES and overall error rates: when the post-error RT in the PoER2 trial was greater than in the PrER trial, overall error rates were lower. Hajcak et al. (2003), for example, correlated post-error RTs with post-error accuracy, and also observed a significant correlation. While different forms of post-error performance parameters were used between the two studies, both studies similarly observed negative correlations between PES and error rates. This confirms that PES functions in a compensatory manner to reduce overall error rates.

Response slowing was also observed in the PoCR2 trial, while PCS was not significantly correlated with commission error rates. This difference between PES and PCS may be consistent with previous findings. Li et al. (2008b) has reported that although post-error and post-correct trials similarly show response slowing, they are differently supported by cortical areas. PES was associated with enhanced activation in the ventrolateral PFC, which is connected with several areas including the SMA within the pMFC (Ide and Li, 2011) and is related to cognitive control. In contrast, PCS was not significantly associated with any brain activation. PCS may reflect automatic delay of sensorimotor transformation or response readiness after inhibition, distinguished from cognitive control *per se* (Li et al., 2005, 2008b). Such differences in background mechanisms for PES and PCS likely manifested themselves differences in response slowing properties related to error recovery in the present study.

### MONITORING AND AFFECTIVELY ORIENTING N2s AT POST-ERROR TRIALS

Comparisons of N2 amplitudes between post-error (PoER1, PoER2) and PrER trials did not yield significant differences, suggesting that neural activities during this time range are modulated by individual differences in behavioral performances and/or behavioral traits. In fact, N2 components for the PoER1 and PoER2 trials were correlated with PES and/or subcomponents of impulsivity traits. We propose that N2 during the PoER2 trial is a monitoring (cognitive control) N2, which contributes to PES and is attenuated by impulsivity. In contrast, N2 during the PoER1 trial may be an orienting N2 affectively enhanced by impulsivity.

N2 for the PoER2 trial was negatively correlated with PES, indicating that more negative, greater N2 activity is associated with greater post-error recovery. This post-error N2 effect is in clear contrast to no significant effect for PCS. As suggested in the literature (MacDonald et al., 2000; Botvinick et al., 2001; Ridderinkhof et al., 2004), N2 during the PoER2 trial likely reflects cognitive control after errors. Chang et al. (2014) also reported that N2 enhancement was observed for PES. This N2 activity may contribute to calming down elevated motor activities, as represented by the negative correlation between the pMFC and motor areas (Danielmeier et al., 2011) or enhanced activation of the ventrolateral PFC connected with the SMA (Li et al., 2008b; Ide and Li, 2011), or may reflect monitoring the conflict between Go and No-go response selections (Botvinick et al., 2001).

The present study also found that N2 activity for the PoER2 trial was particularly attenuated by the affective traits of RR, as demonstrated by the behavioral results. Although the inhibition based model tends to separate motor from reward related impulsivity (impulsive action and impulsive choice, respectively; Bari and Robbins, 2013), motor inhibition affecting PES may be neurally associated with affective reward impulsivity. This intermediary relationship also likely results from neural properties of the pMFC. As argued in the previous study (Ridderinkhof et al., 2004), the pMFC connects several areas such as the ACC, which is probably involved in monitoring N2, as well as the premotor area, and subthalamic nucleus, which is involved in affective and motivational processing (Siegert et al., 2014). The findings suggest that the pMFC coordinates various functions, including post-error control, motor inhibition, and affective arousal (Ridderinkhof et al., 2004). Under the present speeded response conditions, people with greater affective impulsivity may utilize more neural connections with the pMFC for affective processing than during post-error cognitive control, thereby attenuating N2 and its function in monitoring.

The N2 during the PoER1 trial was not significantly correlated with PES, but was negatively correlated with motor impulsivity. Greater motor impulsivity was associated with greater N2 activity, indicating that N2 for the PoER1 trial is different from the monitoring N2. Visual inspection of **Figure 5B** indicates that N2 morphologies differ between the PoER1 and PoER2 trials, suggesting their functional segregation. N2 for the PoER1 trial demonstrated negative deflection until the end of the epoch, while N2 deflection for the PoER2 trial demonstrated convergence, returning to baseline (0 μV) around 300 ms. An interpretation consistent with the behavioral finding that post-error speeding was enhanced by the drive trait is that modulation of N2 for the PoER1 trial is associated with automatic affective orientation promoting negative behaviors.

This interpretation of the N2 effect for the PoER1 trial may also be consistent with the result of the PoCR1 trial. In the absence of errors, the ERP for the PoCR1 was positively correlated with impulsivity trait (NPI), indicating that greater post-correct negativity is associated with smaller impulsive scores in a reverse manner.

Under the present speeded response conditions, errors might strongly elicit affective arousal and enhance rostral ACC activation. Critchley et al. (2005) reported that the ACC is functionally separated into anterior and posterior parts, and the rostral ACC is associated with automatic affective orientation. This rostral ACC activation may be the origin of the orienting N2 during the PoER1 trial, which is modulated by individual differences in motor impulsivity. Conversely, it has also been reported that motor impulsivity can be positively correlated with PFC activation for response inhibition (Goya-Maldonado et al., 2010). Therefore, affective arousal resulting from errors may manifest as individual differences in motor impulsivity, which then may affect inhibitory N2 (Jodo and Kayama, 1992), including during Go trials, while the current speeded conditions did not generally allow the participants to yield PES timely at the PoER1 trial.

### RESPONSE-LOCKED ERROR-RELATED ERPs AT ERROR TRIALS

The present study observed that error trials yielded both ERN/Ne and Pe, consistent with previous studies (Gehring et al., 1993; Nieuwenhuis et al., 2001; Hajcak et al., 2003; Debener et al., 2005). All participants yielded negative potentials corresponding to ERN/Ne (mean: –4.1 μV; range from –1.5 to –8.2 μV), and all but three participants yielded positive potentials for Pe (4.7 μV: range from 0.4 to 9.4 μV), indicating that the participants engaged in error monitoring. However, contrary to previous studies (Gehring et al., 1993; Nieuwenhuis et al., 2001; Hajcak et al., 2003; Debener et al., 2005), we did not find significant relationships between the error ERPs and PES. Although ERN/Ne is not always correlated with PES (Gehring and Fencsik, 2001; Nieuwenhuis et al., 2001; Hajcak et al., 2003; Endrass et al., 2007; Chang et al., 2014), we nonetheless potentially expected a correlation between Pe and PES, which we did not observe in the present study. Such discrepancy between error ERPs and PES may be characteristics of elevated impulsivity. The present study required participants to respond rapidly within a 250 ms deadline, thereby artificially evoking unusual behavioral patterns throughout the experiment. Constant temporal pressure likely evoked an unusual hyper-activation of background neural activities for error ERPs, such as that observed in patients with OCD (Hajcak et al., 2008). Under daily circumstances, larger ERN/Ne activities are responsible for effectively avoiding negative consequences in NH people (Frank et al., 2005). However, if hyper-activation of error related neural activities takes places constantly, it may function as a marker of pathological impulsivity traits (Hajcak et al., 2008), reducing the connection between neural activities and monitoring performance. This possibility implies that long-term exposure to unusual constant speeded circumstances may contribute to unusual mental states comprising pathological impulsivity.

## CONCLUSION

The present speeded behavioral setting has revealed that individual differences in impulsivity traits are associated with changes in post-error monitoring and its related neural activities. Immediately after errors, post-error speeding occurred, and was associated with the drive trait. We have suggested that post-error speeding resulted from non regulatory states resulting from affective excitement or preoccupation evoked by errors. The neural correlate of post-error speeding may be the affective orienting N2, which is enhanced by motor impulsivity. However, PES occurred later than expected, being modulated by the affective impulsive trait of RR. The monitoring N2 contributed to PES, while also being attenuated by RR. This delayed post-error recovery pattern likely originated from unusual neural activities when committing errors. The constant speeded response condition might yield unusual hyper-activation of neural activities for error monitoring, even in healthy people. Future research is needed in order to investigate how error and post-error monitoring occur in clinical populations under similar constant speeded conditions. If some NH individuals produce PES immediately after errors, even under the present experimental condition, further investigation into the types of behavioral traits developed in those individuals may yield advances in knowledge. Further studies investigating whether such behavioral traits are robust against current and/or developing mental illness are required.

## Conflict of Interest Statement

The authors declare that the research was conducted in the absence of any commercial or financial relationships that could be construed as a potential conflict of interest.
